# The Reduction of Traumatic Spinal Cord Secondary Injury by Anti-RhoA siRNA Functionalized Nucleic Acid Nanoparticles (NANPs)

**DOI:** 10.59566/isrnn.2024.0101079

**Published:** 2024

**Authors:** Damian Beasock, Morgan Chandler, Yasmine Radwan, Zhen Liao, Fuying Ma, Ken Webb, Martin Panigaj, Jeoung Soo Lee, Kirill A. Afonin

**Affiliations:** 1 UNC Charlotte, NC, USA;; 2 Drug Design Development and Delivery (4D) Laboratory, Department of Bioengineering, Clemson University, SC 29631, USA;; 3 Microenvironmental Engineering Laboratory, Department of Bioengineering, Clemson University, SC 29631, USA

**Keywords:** Spinal cord injury, NANPs, RhoA/Rho kinase, RNAi

## Abstract

Primary injury of the spinal cord is caused by a mechanical traumatic event which is rapidly followed by a secondary injury cascade that may evolve for several months leading to biological and functional changes. During the secondary injury, many pathophysiological pathways and process are activated including inflammation, oxidative stress, demyelination, excitotoxicity, axon degeneration, and cell death. The RhoA/Rho kinase pathway significantly contributes to spinal degeneration and regeneration and therefore represents a potential therapeutic target. Nucleic acid nanoparticles (NANPs) offer easy rational and programable design with the potential to carry on multiple synergistic therapeutic nucleic acid functional motifs. In this context we designed, synthesized, and assembled several representative NANPs decorated with multiple copies of siRNAs targeting RhoA. Subsequently we assessed NANPs’ physicochemical properties, toxicity, and immunorecognition upon delivery with the nanocarrier PLGA-g-PEI (PgP), developed with the aim to select for the most immunoquiescent type of formulations. In addition, we observed that L1 neural cell adhesion molecule conjugated PgP (L1-PgP) efficiently delivered NANP-siRhoA in cultured neuroblastoma (B35) cells. RhoA mRNA expression was significantly reduced by all L1-PgP/ NANP-siRhoA relative to the untreated control, while no significant differences were observed between the different NANP-siRhoAs.

## INTRODUCTION

Spinal cord injuries (SCI) are one of the most serious traumatic events leading to significant decrease in quality of life. Generally, they may be caused by external physical force or by endogenous non-traumatic pathological processes such as tumor or infection. Traumatic injury causes structural changes to the spine that initiate complex secondary processes resulting in tissue damage and its poor or lack of regeneration, which ultimately leads to interrupted neuronal communication^1^. The secondary injury is a complex long-term process spanning from weeks to months depending on the initial traumatic impact. The extent and severity of the initial trauma are key determinants in shaping the required therapeutic interventions. In the complex scheme of molecular interactions involved in pathology or regeneration of the spine, the Ras homolog family member A (RhoA) protein is one of the significant players^2^. In neurons, RhoA induces axonal and dendritic retraction and spine/synapse loss^3^. The activation and expression of RhoA highly increases immediately after SCI and in the days/weeks following the injury^2, 4, 5^. The elevated production has been associated with inflammation following the SCI event. Although secondary injury involves a complex system of cascades, previous studies have shown that inhibition of RhoA with antagonists, C3 transferase^2^, siRNA targeting RhoA^6–12^, or genetic ablation^6^ can reduce secondary injury.

**Fig. 1.**
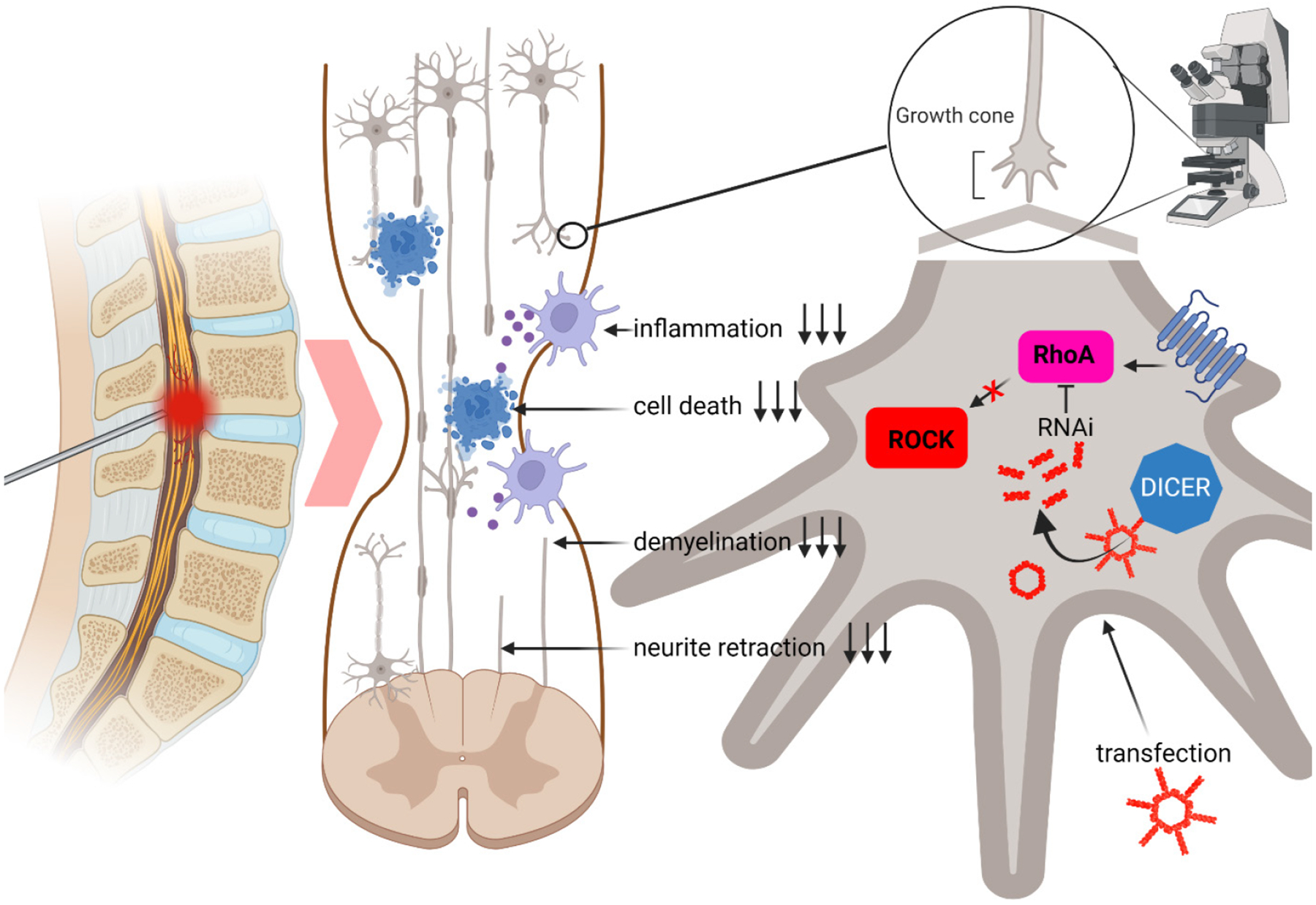
The expression of RhoA increases within less than two hours after mechanical injury of the spinal cord and remains elevated for several months as observed in rat and murine models. The RhoA/Rho kinase pathway contributes to several adverse pathogenic conditions^2^. Downregulation of RhoA is one of the possible therapeutic applications. Herein, we attempted to deliver siRNAs targeting RhoA embedded in NANP structures (NANP-siRhoA) or as a simple duplex (for simplicity, only ring NANPs are depicted).

Nucleic acid nanoparticles, or NANPs, are a new complex class of therapeutic nucleic acids that exhibit unique and tunable physicochemical properties, biological activities, and interactions with the immune system. Depending on the composition, dimensionality, and sequence, NANPs can be immunoquiescent or stimulate a specific immune response while simultaneously carrying out multiple therapeutic functions^7, 8^. In our previous studies, we systematically examined those immunostimulatory properties and described general trends that render NANPs immunostimulatory or immunoquiescent^8^, reviewed in^9,10^. With this knowledge, several NANP types have been designed to carry multiple copies of Dicer substrate (DS) RNAs which upon intracellular dicing, release siRNAs targeting RhoA (Fig. 1). The formation of functional NANPs has been confirmed by EMSA and AFM experiments and all NANPs have been examined in several immune reporter cells to assess their immunorecognition. Since the inflammation during the secondary injury after SCI is detrimental for spinal cord healing, we hypothesized that the least immunogenic NANPs will serve as the best scaffold for the delivery of RhoA siRNAs. In addition, naked NANPs without a carrier are invisible to cells and cannot enter the cytoplasm^8^. This issue complicates the delivery and immunoquiescence of NANP-siRhoA, as we know that complexation of NANPs with different carriers can alter the immunomodulatory properties of transfected NANPs^11, 12^. Therefore, we evaluated the knockdown efficacy of various NANP-siRhoA with a novel cationic amphiphilic copolymer micelle carrier, poly (lactide-co-glycolide)-graft-polyethylenimine (PgP, US Patent 10,232,050) developed by Jeoung Soo Lee^12–17^ and compared to widely used commercial Lipofectamine^™^ 2000 (L2K) in various reporter cell lines *in vitro*. PgP can deliver NANP-siRhoA in a wide range of neural cell types, but neurons remain the most challenging target cells for the TNA delivery and the most important therapeutic target for functional recovery. To overcome this, L1 neural cell adhesion molecule as a neuronal targeting moiety has been selected based on its endogenous up-regulation following axotomy^18, 19^ and ability to promote neurite outgrowth^20, 21^, neuronal survival^22^, and axonal regeneration and functional recovery in animal SCI models^23, 24^. Webb’s lab has previously shown that substrates with covalently immobilized L1 selectively support adhesion of neurons from mixed cell suspensions including astrocytes and meningeal fibroblasts^25^. L1 exhibits homophilic binding and is endocytosed at the rear of growth cones and recycled to the leading edge^26–28^. Collectively, these previous studies suggest that L1 offers a ligand for neuron-specific targeting and improved endocytic uptake, as well as potential bioactivity for improving neuronal survival and axonal regeneration. Therefore, we conjugated L1 neural cell adhesion molecule to the PgP (L1-pgP) surface and evaluated the knockdown efficiency of NANP-siRhoA delivered with L1-PgP to increase selectivity for neurons.

## MATERIAL AND METHODS

### Synthesis and characterization of various functional NANP-siRhoAs

Several representative NANPs (RNA rings^29^, RNA cubes^30^, and RNA fibers^31^) were rationally designed^32^ to assemble into NANPs functionalized with the DS RNAs designed to target RhoA mRNA (Gene ID: 117273). All RNA sequences are listed in the supporting information. The corresponding DNA template strands, forward, and reverse primers were purchased from Integrated DNA Technologies (Coralville, IA, USA). The templates were amplified by PCR using 2X MyTaq^™^ Mix (Bioline, London, UK). Purified templates by DNA Clean and Concentrator^™^ kit from Zymo Research (Irvine, CA, USA) were transcribed by *in vitro* run-off transcription using T7 RNA polymerase in the presence of 80 mM HEPES-KOH (pH 7.5), 2.5 mM spermidine, 50 mM DTT, 25 mM MgCl_2_, and rNTPs (25 mM/each). After approximately 3.5 hours of incubation at 37 °C, the RNA was incubated with RQ1 RNase-free DNase (New England BioLabs, Ipswich, MA, USA) for 30 minutes at 37 °C. The resulting RNA strands were purified via 8 M urea polyacrylamide gel electrophoresis (PAGE) in 89 mM tris-borate, 2 mM EDTA buffer (pH 8.2). The RNA strands were visualized using UV shadowing and RNA was washed out from excised gel fragments after overnight incubation in 300 mM NaCl, 89 mM tris-borate, and 2 mM EDTA (pH 8.2). Subsequently, the RNA was precipitated in approximately 2.5 volumes of 100% ethanol at −20 °C for 3 hours. Samples were centrifuged and rinsed with 90% ethanol, followed by vacuum drying and resuspension in double-deionized endotoxin-free water. Owing to the efficient design, all RNA strands across multiple batches have been of high yield and purity, as assessed via absorbance measured using a NanoDrop 2000 spectrophotometer. NANPs were assembled in one-pot reactions from individual RNA strands in equimolar ratio. For RNA rings and RNA fibers, the samples were heated to 95 °C for 2 minutes, snap-cooled to 4 °C for 2 minutes, and assembly buffer (89 mM tris-borate (pH 8.2), 2 mM MgCl_2_) was added prior to incubation at 30 °C for 30 minutes. For the RNA cubes, the samples were heated to 95 °C for 2 minutes, incubated at 45 °C for 2 minutes, and assembly buffer was added prior to further incubation at 45 °C for 20 minutes. In addition, DS RhoA was prepared by mixing the sense and antisense RNAs targeting RhoA mRNA in an equimolar ratio. The sample was heated to 95 °C for 2 minutes and assembly buffer was added prior to further incubation at room temperature for 20 minutes as the duplex annealed. All assemblies have been confirmed using 8% non-denaturing native-PAGE (37:5:1) in the presence of 89 mM tris-borate (pH 8.2) and 2 mM MgCl_2_. Native-PAGEs were run for 20 minutes at 300 volts on a Mini-PROTEAN^®^ Tetra system by Bio-Rad (Hercules, CA, USA) at 4 °C. Assemblies were visualized by total-staining with ethidium bromide and recorded by ChemiDoc MP (Bio-Rad). The presence of a single distinct band in the gel indicated the assembly of the defined nanoconstruct. NANPs’ integrity and dimensionality was confirmed by atomic force microscopy (AFM) as detailed in previous works^33, 34^. Imaging was done in tapping mode on a MultiMode AFM Nanoscope IV system (Bruker Instruments, Santa Barbara, CA, USA).

### Cell culture and transfection

Immunostimulatory properties of individual NANPs were assessed in reporter cell lines (InvivoGen). The following cells each overexpressing a particular nucleic acid-specific pattern recognition receptor with downstream reporters for assessing activation were used: HEK-Blue^™^ hTLR7, HEK-Lucia^™^ RIG-I, and THP1-Dual cells. Cells were maintained according to the manufacturer’s recommendations. The growth medium for all HEK cells was DMEM, 4.5 g/L glucose, 10% (v/v) heat-inactivated fetal bovine serum, 100 U/mL penicillin, 100 μg/mL streptomycin, 100 μg/mL Normocin^™^, and 2 mM L-glutamine, with the addition of selective antibiotics- the HEK-Blue^™^ Selection mix or blasticidin and zeocin^™^. All cell lines were used at low passage numbers to ensure integrity of the engineered expression system.

All cells were plated in flat-bottom 96-well plates at a density of 40×10^3^ cells/well 24 hours prior to all experiments and were maintained in incubators at 37 °C and 5% CO_2_. Assembled NANP-siRhoAs were delivered into the cells either in complex with PgP (in amounts based on the previously established N:P ratio of 30/1)^13^ or with L2K as a control and were incubated at 37 °C for 30 minutes. Throughout all experiments, the amounts of nanostructures carrying the therapeutic DS RhoAs were kept constant at 10 nM final concentration, while the amounts of PgP varied. All functional NANPs and control DS RhoA duplexes were added to the plates using either PgP or L2K as carriers; all samples were added to the plates in triplicates. As controls, a panel of known positive controls per each cell line were utilized for all reporter cells, including 3p-hpRNA (10 ng/mL), R848 (2 μg/mL), 2’3’-cGAMP (2 μg/mL), and Poly I:C (2 μg/mL) at concentrations based on the manufacturer’s recommendations and in triplicate wells. For 3p-hpRNA which requires a carrier, L2K and PgP were both used for comparison. 24 hours after transfection, QUANTI-Blue^™^ solution from InvivoGen was used to assess the level of secreted embryonic alkaline phosphate activity in all HEK-Blue^™^ cell lines and THP1-Dual cells. 180 μL of the QUANTI-Blue^™^ solution was added with 20 μL of cell supernatant from the transfected plates of reporter cells in a fresh flat-bottom 96-well plate for incubation at 37°C and 5% CO_2_. After approximately 3 hours of incubation, the QUANTI-Blue^™^-treated plates were read on a Tecan Spark plate reader at 638 nm. Well values were the averages of sixteen-point reads. Alternatively, for the HEK-Lucia^™^ and THP1-Dual cell lines, QUANTI-Luc^™^ solution from InvivoGen was used to assess the activity of Lucia luciferase as a reporter. 50 μL of QUANTI-Luc^™^ and 20 μL of cell supernatant were added to a black-walled and flat-bottomed 96-well plate which was then read immediately for luminescence using a Tecan Spark plate reader. For analysis, the triplicate technical repeat wells of each plate were averaged into one biological repeat. Five separate biological repeats were repeated for each cell line for each DS RhoA nanostructure. All averages were normalized to cells-only and represented as normalized fold induction.

After analysis of the cell supernatant, the remaining transfected cells were assessed for cell viability to determine the overall toxicity of the DS RhoA/PgP polyplexes in the reporter cell lines. CellTiter 96^®^ AQueous One Solution (MTS) was used to assess cell viability in each cell line. To each 100 μL of cells per well, 20 μL of the MTS solution was added, followed by incubation at 37°C and 5% CO_2_ for 75 minutes. Afterwards, the plates were read on a Tecan Spark plate reader at an absorbance of 490 nm. Well values were the averages of sixteen-point reads. All averages were normalized to cells-only and represented as normalized percent cell viability.

### Preparation of L1-PgP

**Fig. 2.**
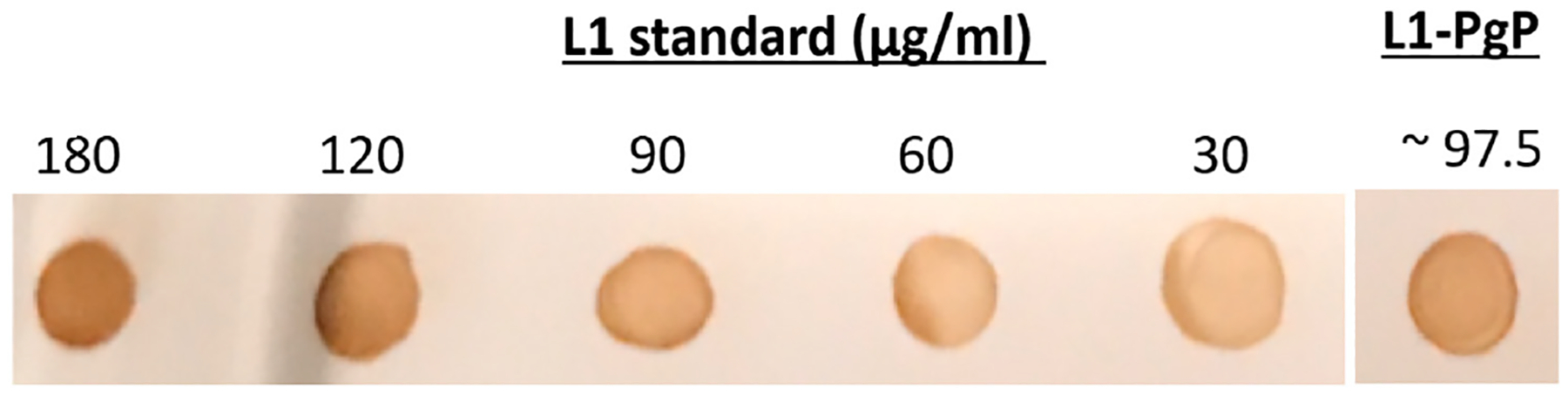
Immunoblot assay using L1 antibody after L1 conjugation to PgP (1:3 mole ratio).

Recombinant L1 was purified from High 5 insect cells transfected with baculoviral vector by Dr. Webb and the purified L1 was conjugated to PgP using 1-ethyl-3-(dimethylaminopropyl) carbodiimide (EDAC) and N-hydroxysuccinimide (NHS) with a ratio of 1:3 mole L1:PgP. The conjugation of L1 was verified by immunoblot using primary antibody against L1 (Fig. 2). The amount of L1 conjugated to PgP determined by ELISA and the amount of L1 conjugated to PgP determined by ELISA was ~97.5 μg L1/mg PgP.

### Characterization of L1-PgP/NANP-siRhoA polyplex

To evaluate the L1 conjugation to PgP upon formation of the polyplex, L1-PgP/RhoA-NANP at an N/P ratio of 30/1 were prepared and polyplexes were electrophoresed on a 2% (w/v) agarose gel containing ethidium bromide (0.5 μg/ml) for 90 min at 80V. In order to evaluate the ability of L1-PgP to protect NANP-siRhoA, L1-PgP/NANP-siRhoA was incubated with heparin (heparin: NANP-siRhoA at a =3:1 w/w ratio) for 30 minutes at 37 °C and then electrophoresed. The gel was imaged on a UV illuminator (ChemiDoc-IT^2^ Imager, UVP) to visualize the migration of polyplexes and naked siRhoA (silencer^®^ Pre-designed siRNA targeting RhoA, Ambion).

### Knockdown efficiency of L1-PgP/NANP-siRhoA polyplexes

Neuroblastoma (B35, 8×10^4^ cells/well) cells were seeded in 24-well plates using DMEM/F12 supplemented with 10% FBS and 100 IU/ml penicillin/100 μg/ml streptomycin. After overnight incubation, the cells were washed twice with fresh media containing 10% serum. The cells were transfected with various L1-PgP/NANP (1 μg siRhoA) polyplexes at an N/P ratio of 30/1 in medium containing 10% FBS, incubated for 24 hours, and then the medium containing polyplexes was removed and replaced with fresh medium containing 10% FBS. The cells were incubated an additional 48 hours. PgP/ DS RhoA was also included for comparison. Non-transfected cells were used as a control. The cells were transfected with polyplexes. At 72 hours post-transfection, the cells were lysed, and total RNA was isolated using RNeasy mini kit. The isolated RNA quality and quantity were evaluated by Take 3 using a BioTek synergy microplate reader (BioTek, Synergy HT). Complementary DNA (cDNA) was synthesized by reverse transcription reactions with isolated total RNA (0.5 μg) using MultiScribe^™^ reverse transcriptase with random primers (High Capacity cDNA Reverse Transcription Kit; Ambion applied biosystem). Real-time PCR was performed using target-specific primers (final concentration: 0.5 μM) using SYBR Green PCR kit in a Rotorgene Q thermal cycler (Qiagen). Glyceraldehyde-3-phosphate dehydrogenase (GAPDH) was used as an endogenous control. Primers for Rho A: forward primer: 5’-CAA GGA CCA GTT CCC AGA GG -3’; reverse primer: 5’-GCT GTG TCC CAT AAA GCC AAC -3’. Primers for GAPDH: forward primer: 5’- ATG GCC TTC CGT GTT CCT AC-3’; reverse primer: 5’-TAG CCC AGG ATG CCC TTT AG-3’. The cycle number at which the amplification plot crosses the threshold was calculated (CT). Relative mRNA expression levels of RhoA were calculated using the 2^−ΔΔCt^ method^35^. The minus RT (reverse transcriptase) reactions performed on a representative subset of samples demonstrated that genomic DNA contamination was not significant (data not shown). Reaction specificities were routinely verified by melting curve analysis.

### Statistical analysis

Statistical analysis was performed using one-way analysis of variance (ANOVA) with multiple post-hoc comparison Tukey test to determine significant difference between groups using GraphPad. version 10.0.0 for Windows, GraphPad Software, Boston, Massachusetts USA, www.graphpad.com.

## RESULTS AND DISCUSSION

**Fig. 3.**
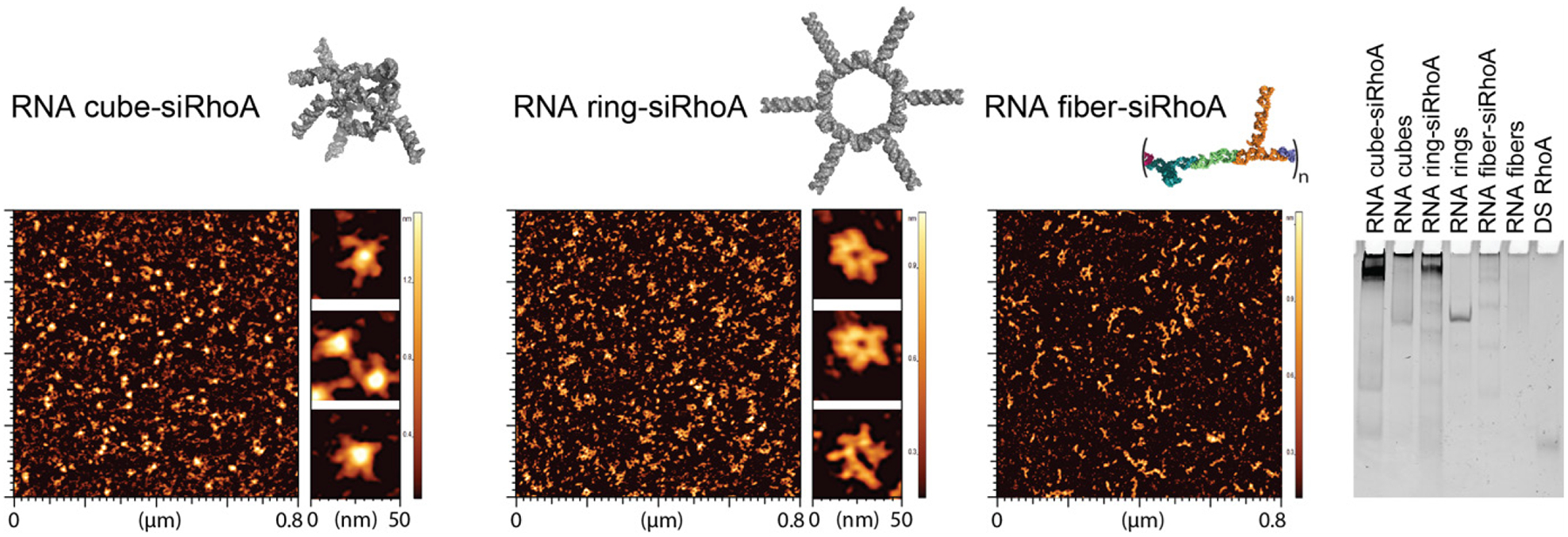
Design and characterization of different NANP-siRhoAs. Representative 3D models, AFM images, and native-PAGE results show the DS RhoA functionalized RNA cubes, RNA rings, and RNA fibers.

The structures of individual NANPs functionalized with DS RNAs targeting RhoA mRNA were confirmed using AFM and compared to nonfunctionalized RNA NANP scaffolds. AFM images of NANP-siRhoAs revealed that the assemblies maintained a uniform size and shape, resembling the established non-functionalized scaffolds^36^. Additionally, differences in migration between functionalized and non-functionalized NANPs were observed through native-PAGE analysis (Fig. 3).

**Fig. 4.**
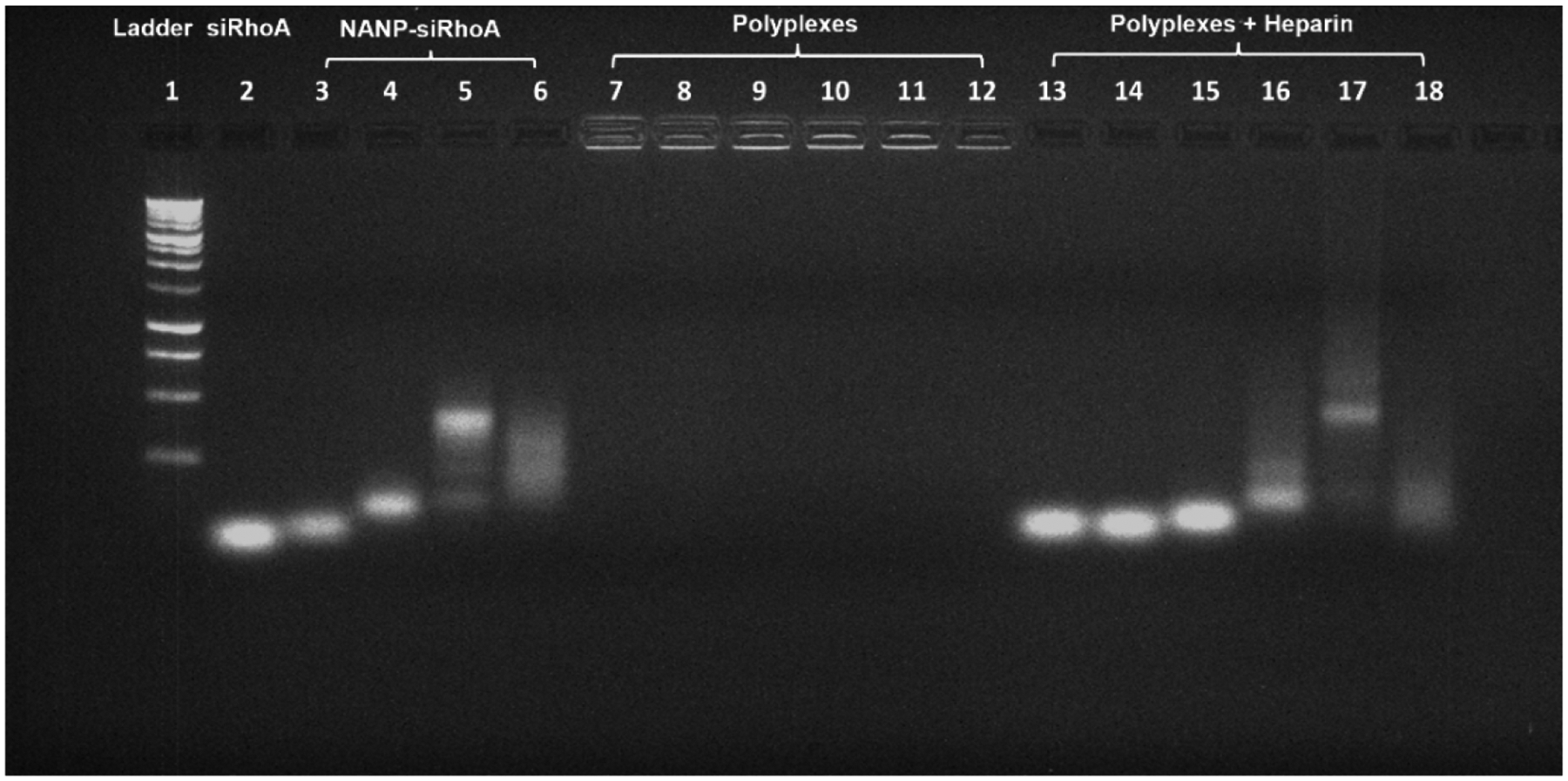
Complex formation and stability of L1-PgP/NANP-siRhoA polyplexes by agarose gel electrophoresis and integrity of NANP-siRhoAs by heparin competition assay. Lane 1: molecular wight marker, 2: naked siRhoA, 3–6: NANP-siRhoAs (3: duplex, 4: RNA ring-siRhoA, 5: RNA cube-siRhoA, 6: RNA fiber-siRhoA), 7: PgP/siRhoA, 8: L1-PgP/siRhoA, 9–12: L1-PgP/NANP-siRhoA polyplexes (9: L1-PgP/duplex, 10: L1-PgP/RNA ring-siRhoA, 11: L1-PgP/RNA cube-siRhoA, 12: L1-PgP/RNA fiber-siRhoA), 13–18: dissociated siRNAs from polyplexes after heparin treatment (13: PgP/siRhoA, 14: L1-PgP/siRhoA, 15: L1-PgP/duplex, 16: L1-PgP/RNA ring-siRhoA, 17: L1-PgP/ RNA cube-siRhoA, and 18: L1-PgP/RNA fiber-siRhoA)

The stable polyplex formation of L1-PgP with various NANP-siRhoA was confirmed with gel retardation assays using PgP/siRhoA and L1-PgP/siRhoA for comparison (Fig. 4). We observed complete retardation of electrophoretic mobility for all the L1-PgP/ NANP-siRhoA polyplexes (column 9–12) as shown for PgP/siRhoA (column 7) and L1-PgP/siRhoA (column 8). We also observed that intact NANP-siRhoAs (column 15–18) were detected after dissociation by heparin as shown for PgP/siRhoA (column 13) and L1-PgP/siRhoA (column 14). Therefore, we demonstrated that L1-conjugation to PgP did not affect the formation of polyplexes with siRhoA or the various NANP-siRhoAs.

**Fig. 5.**
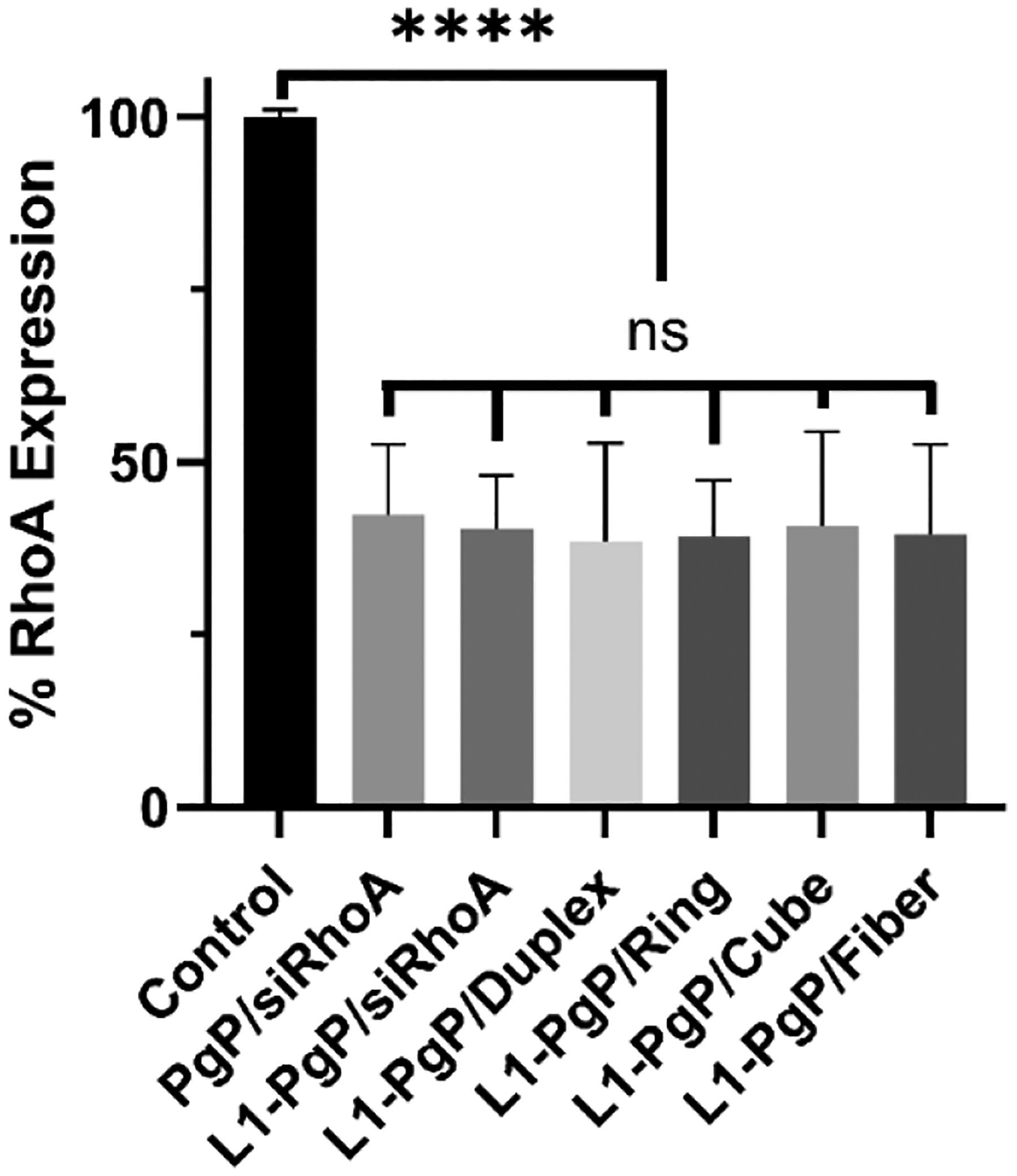
RhoA knockdown efficiency was assessed following the transfection of various L1-PgP/NANP-siRhoA polyplexes at an N/P ratio of 30/1 in neuroblastoma (B35) cells cultured in media containing 10% serum. At 72 hours post-transfection, RhoA expression levels were measured by RT-PCR. Data are presented as mean ± SD (n=3). Statistical significance is denoted as ****: P < 0.001 compared to the control; ns: not significant. (Key: Duplex – DS RhoA; Ring – RNA ring-siRhoA; Cube – RNA cube-siRhoA; Fiber – RNA fiber-siRhoA).

To evaluate the knockdown efficiency of L1-PgP/NANP-siRhoA polyplexes, L1-PgP/NANP-siRhoA polyplexes at an N/P ratio of 30/1 were prepared and transfected in B35 cells in media containing 10% serum. PgP/siRhoA and L1-PgP/siRhoA polyplexes were prepared at an N/P ratio of 30/1 and used for comparison. RT-PCR showed that RhoA mRNA expression was significantly decreased for all transfected L1-PgP/NANP-siRhoA polyplexes compared to the untransfected control group, while it was not significantly different between the NANP-siRhoAs nor PgP/siRhoA (Fig. 5).

Activation of the innate immune system during post-traumatic recovery of the spinal cord can interfere with wound healing. Therefore, the complexation of NANP-siRhoAs with a carrier should ideally be immunoquiescent. To assess the immunostimulatory properties of NANPs, we first examined the predominant final effector triggered by immune stimulation in the THP1 Dual cell line. This cell line is engineered to express secreted alkaline phosphatase (SEAP) when activated pathways converge on the nuclear factor kappa-light-chain-enhancer of activated B cells (NF-κB), or luciferase when signaling pathways merge on interferon-regulatory factor (IRF)-regulated transcription.

**Fig. 6.**
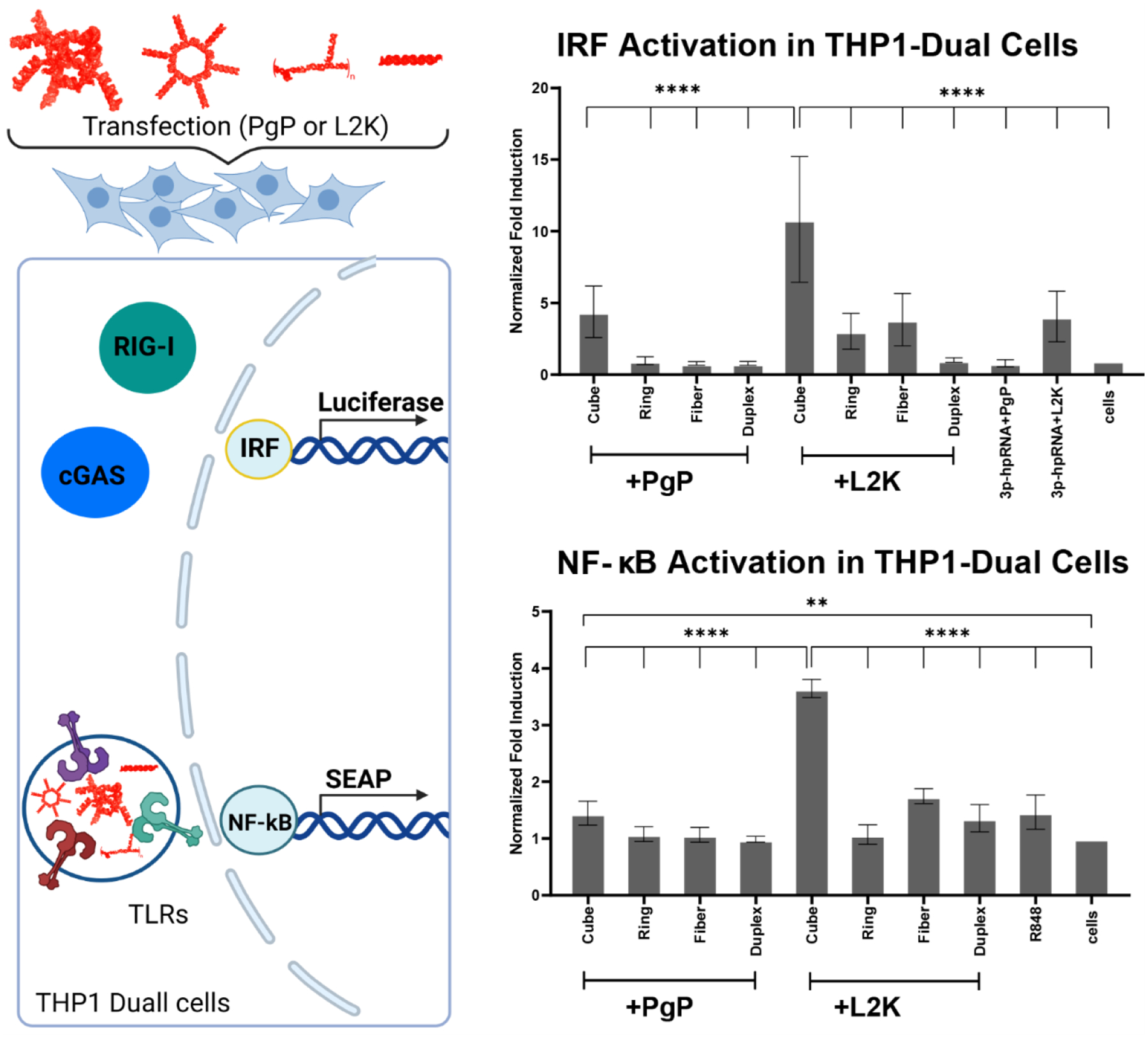
Normalized induction of IRF and NF-κB immune pathways in the recognition of NANP-siRhoAs delivered with PgP versus L2K. Means are shown of n=5 biological replicates, each the average of three technical repeats. Error bars denote SD. (Key: Duplex – DS RhoA; Ring – RNA ring-siRhoA; Cube – RNA cube-siRhoA; Fiber – RNA fiber-siRhoA).

All NANP-siRhoAs transfected with PgP induced weaker immune responses in THP1 Dual cells compared to those complexed with L2K (Fig. 6). Planar rings and fibers showed low immunostimulatory activity when associated with PgP, but not when complexed with L2K. In contrast, cube RNAs elicited the strongest response with L2K, though their transfection with PgP still significantly enhanced the activation of both NF-κB and IRF pathways.

These findings suggest that planar NANPs complexed with PgP represent a more immunoquiescent system, making them a promising therapeutic option for addressing secondary injuries after spinal cord injury (SCI), as inflammation inhibits the healing process.

**Fig. 7.**
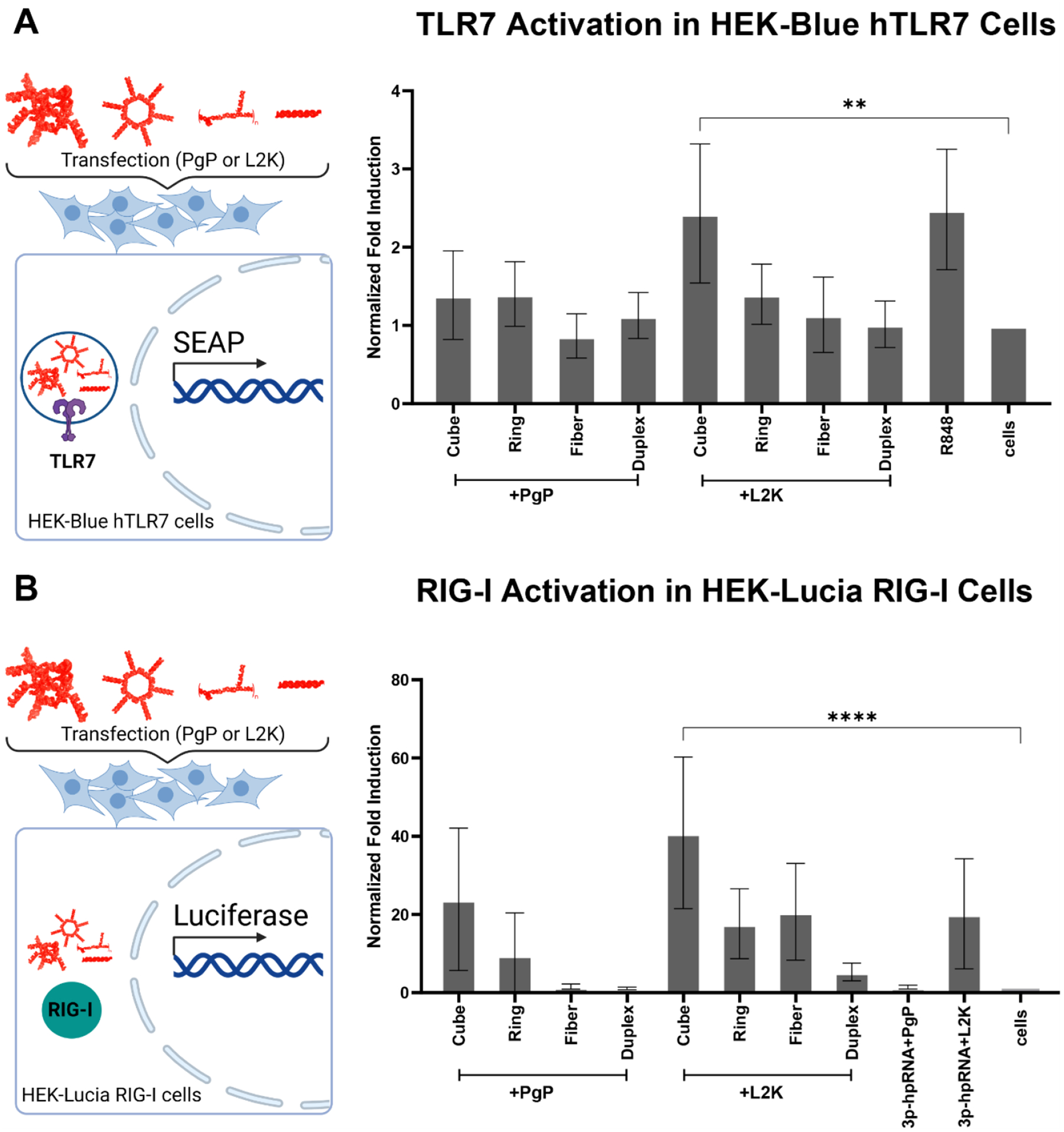
Normalized immunostimulation in reporter cells of NANP-siRhoAs delivered using PgP versus L2K. (Key: Duplex – DS RhoA; Ring – RNA ring-siRhoA; Cube – RNA cube-siRhoA; Fiber – RNA fiber-siRhoA). (**A**) Cell lines produce SEAP downstream of hTLR7 expression. Means are of n=5 biological replicates, each the average of three technical repeats. Error bars denote SD. The positive control shown is R848 for hTLR7. (**B**) Normalized immunostimulation in reporter HEK-LUCIA^™^ RIG-I cells of NANP-siRhoAs delivered using PgP versus L2K. Cell lines produce Luciferase downstream of HEK- RIG-I expression. Means are of n=5 biological replicates, each the average of three technical repeats. Error bars denote SD. The positive control shown is 3p-phRNA.

During transfection, the first pattern recognition receptors (PRRs) to encounter delivered nucleic acids are Toll-like Receptors (TLRs 3, 7, 8, and 9). In TLR7 HEK reporter cells, we observed immune pathway activation only when functional RNA cubes were delivered with L2K, but not with PgP. Transfections with other structures produced signals similar to those of untreated cells, regardless of the transfection agent used (Fig. 7A).

Because our constructs were prepared by run-off in vitro transcription, the resulting RNA strands contained triphosphates on their 5′ ends, which are known to strongly activate the cytosolic RNA sensor RIG-I^34, 37–40^. However, transfection of NANP-siRhoAs activated RIG-I only when delivered by L2K. Some activation was also observed for RNA cubes formulated with PgP, but the variability in the data prevents definitive conclusions. Interestingly, delivery of the positive control for RIG-I (3p-hpRNA) using PgP versus L2K showed a notable difference: 3p-hpRNA delivered by PgP was almost unrecognizable by RIG-I (Fig. 7B).

**Fig. 8.**
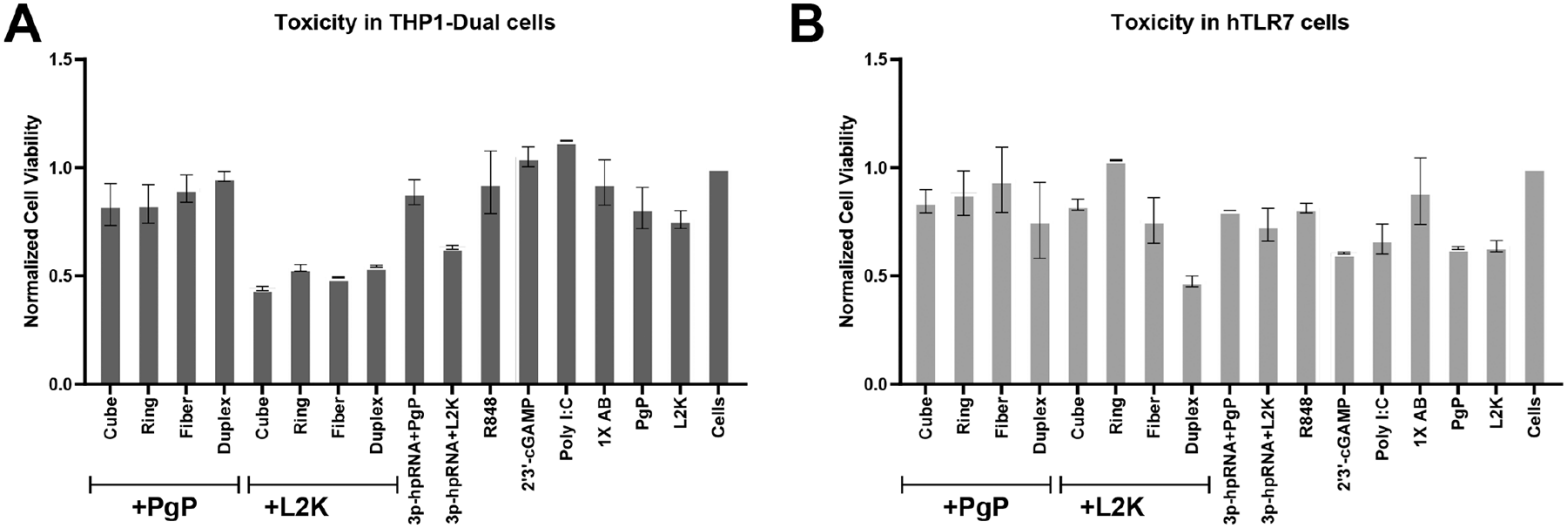
Cytotoxicity of (**A**) THP1-Dual and (**B**) HEK-Blue hTLR7immune reporter cells after transfection with NANP-siRhoAs delivered with PgP versus L2K. Error bars denote SD of n=3 wells. (Key: Duplex – DS RhoA; Ring – RNA ring-siRhoA; Cube – RNA cube-siRhoA; Fiber – RNA fiber-siRhoA)

Cell viability assays in THP1 Dual cells revealed an approximately 50% reduction in viability when NANP-siRhoAs were complexed with the L2K carrier (Fig. 8A). In contrast, no significant changes in cell viability were observed in hTLR7-expressing cells (Fig. 8B), irrespective of the type of NANP used (except for the duplex) or the transfection agent.

In conclusion, we identified a NANP-siRhoA candidate which, in complex with PgP, has minimal immunostimulatory properties in investigated reporter cell lines. In addition, we demonstrated that NANP-siRhoAs delivered with L1-PgP can effectively downregulate RhoA expression upon delivery to a neuroblastoma cell line. Overall, our data suggest the feasibility of NANP-siRhoAs for therapeutic potential in the future treatment of spinal cord injury and confirm the ability of NANP-siRhoAs to deliver multiple copies of therapeutic nucleic acids. The developed platform can be further investigated for a broad range of biomedical applications.

## Supplementary Material

1
