# Supplementary material for: The Reduction of Traumatic Spinal Cord Secondary Injury by Anti-RhoA siRNA Functionalized Nucleic Acid Nanoparticles (NANPs)
Source: RNA Nanomed. Author manuscript; Available in PMC 2025 Apr 30. (PMC12043328; doi:10.59566/isrnn.2024.0101079)
Supplement: 1 [file NIHMS2068822-supplement-1.pdf]

SUPPORTING INFORMATION

RNA sequences of NANPs monomers

<u>RNA cube-siRhoA</u>

A 5’- *GGCAACUUUGAUCCCUCGGUUUAGCGCC GGCCUUUUCUCCCACACUUUCACGUUCC UGCUUCAUUUUGGCUAACUCCCGCCUU* -3’

B 5’- *GGAAAUUUCGUGGUAGGUUUUGUUGCC CGUGUUUCUACGAUUACUUUGGUCUUCC UGCUUCAUUUUGGCUAACUCCCGCCUU* -3’

C 5’- *GGACAUUUCGAGACAGCAUUUUUUCCC GACCUUUGCGGAUUGUAUUUUAGGUUCC UGCUUCAUUUUGGCUAACUCCCGCCUU* -3’

D 5’- *GGCGCUUUUGACCUUCUGCUUUAUGUCC CCUAUUUCUUAAUGACUUUUGGCCUUCC UGCUUCAUUUUGGCUAACUCCCGCCUU* -3’

E 5’- *GGGAGAUUUAGUCAUUAAGUUUUACAAU CCGCUUUGUAAUCGUAGUUUGUGUUUCC UGCUUCAUUUUGGCUAACUCCCGCCUU* -3’

F 5’- *GGGAUCUUUACCUACCACGUUUUGCUGU CUCGUUUGCAGAAGGUCUUUCCGAUUCC UGCUUCAUUUUGGCUAACUCCCGCCUU* -3’

RhoA sense 5’- *pGGCGGGAGUUAGCCAAAAUGA AGCAGG* -3’

<u>RNA ring-siRhoA</u>

A 5’- *GGGAAUCCGUCCACUGGAUUCCCGUCAC AGAGCCUGCCUGUGACUUCCUGCUUCAU UUUGGCUAACUCCCGCCUU* -3’

B 5’- *GGGAAUCCGCAGGCUGGAUUCCCGUCAC AGAGAACGCCUGUGACUUCCUGCUUCAU UUUGGCUAACUCCCGCCUU* -3’

C 5’- *GGGAAUCCGCGUUCUGGAUUCCCGUCAC AGACGUCUCCUGUGACUUCCUGCUUCAU UUUGGCUAACUCCCGCCUU* -3’

D 5’- *GGGAAUCCGAGACGUGGAUUCCCGUCAC AGUCGUGGUCUGUGACUUCCUGCUUCAU UUUGGCUAACUCCCGCCUU* -3’

E 5’- *GGGAAUCCACCACGAGGAUUCCCGUCAC AGAACCAUCCUGUGACUUCCUGCUUCAU UUUGGCUAACUCCCGCCUU* -3’

F 5’- *GGGAAUCCGAUGGUUGGAUUCCCGUCAC AGAGUGGACCUGUGACUUCCUGCUUCAU UUUGGCUAACUCCCGCCUU* -3’

RhoA sense 5’- *pGGCGGGAGUUAGCCAAAAUGA AGCAGG* -3’

<u>RNA fiber-siRhoA</u>

A 5’- *GGGAAUCCAAGGAGGCAGGAUUCCCGUC ACAGAAGGAGGCACUGUGAC* -3’

B 5’- *GGGAACGUAAGCCUCCAACGUUCCCGGA UGCUAAGCCUCCAAGCAUCCUUCCUGCU UCAUUUUGGCUAACUCCCGCCUU* -3’

RhoA sense 5’- *pGGCGGGAGUUAGCCAAAAUGA AGCAGG* -3’

<u>DS RhoA duplexes</u>

RhoA sense 5’- *pGGCGGGAGUUAGCCAAAAUGA AGCAGG* -3’

RhoA antisense 5’- *CCUGCUUCAUUUUGGCUAAC UCCCGCCUU* -3’
